# Research Progress in Field Grading Materials for New Power Systems

**DOI:** 10.3390/molecules31122021

**Published:** 2026-06-09

**Authors:** Peng Han, Zheng Zhang, Jiayang Li, Geng Li, Hailong Zhang, Yurong Shi, Kehan Xu, Shiquan Guo, Dongli Zhang, Chen Zhao

**Affiliations:** 1 School of Materials Science and Engineering, North China University of Water Resources and Electric Power, Zhengzhou 450045, China; 2 School of Electrical and Information Engineering, Zhengzhou University of Light Industry, Zhengzhou 450002, China; 3 Academy of Agricultural Planning and Engineering, Key Laboratory of Technologies and Models for Cyclic Utilization from Agricultural Resources, Ministry of Agriculture and Rural Affairs, Beijing 100125, China

**Keywords:** new power systems, field grading materials, nonlinear conductivity, thermal conductivity, cable accessories, thermochromic materials

## Abstract

With the rapid construction of new power systems characterized by high renewable energy penetration, high power electronics integration, and high voltage levels, the insulation reliability of critical power equipment—including cable accessories, gas-insulated switchgear (GIS), and power electronic modules—faces unprecedented challenges. Field grading materials (FGM), as core functional media for adaptive electric field homogenization and insulation failure prevention, have emerged as a research hotspot spanning materials science, electrical engineering, and polymer engineering. Starting from the current research status of FGM, this review systematically summarizes filler optimization strategies, covering single fillers, hybrid fillers, trace co-fillers, and structural modification approaches. The applications of FGM in transmission cables, GIS, high-voltage electrical machines, and wide-bandgap power electronic modules are then elaborated in detail. Emphasis is placed on performance enhancement routes of FGM, particularly thermal conductivity improvement via constructing three-dimensional thermally conductive networks and intelligent early warning based on thermochromic materials. Finally, the existing bottlenecks of FGM are analyzed in terms of material stability, multi-physical field coupling adaptation, and engineering industrialization. Future development trends are prospected toward high-performance, multifunctional, intelligent, and engineering-oriented FGM. This review aims to provide theoretical references and technical support for the design and application of advanced FGM in new power systems.

## 1. Introduction

Guided by the “carbon peaking and carbon neutrality” goals, the energy structure of power systems is progressively transitioning from fossil fuels to renewable energy sources such as wind and photovoltaic power as the mainstay. The high penetration of renewable energy and power electronic equipment into the grid has significantly increased the volatility, randomness, and intermittency of the power supply side, while substantially reducing system inertia. Consequently, the stability of grid frequency and voltage faces considerable challenges [1,2].

Constrained by manufacturing and transportation lengths, cable segments must be connected via joints, and terminations are required to link them to equipment such as transformers and electrical appliances. Statistics show that in high-voltage direct-current transmission systems, cable accessory failures account for 70% of line faults [3], with insulation failures making up as high as 97% of these [4]. The root cause lies in the long-term operation of cable accessories in complex environments characterized by the coupling of multiple physical fields such as electric, thermal, and mechanical stress fields. This leads to irreversible deterioration of the dielectric properties of the accessory insulation materials, resulting in overheating of the cable accessories and ultimately causing dielectric breakdown or even fire [5]. Simultaneously, high-voltage output and frequent thermal cycling in power systems are the core issues leading to heat accumulation within cable accessories. For electronic devices, each 2 °C rise in temperature reduces reliability by 10% [6]. For transformer windings, every 6 °C increase in temperature cuts the expected lifespan by 50% [7,8,9,10,11].

As critical components within cable accessories, field grading materials (FGM) have attracted significant research attention [12]. We have systematically summarized relevant classical theories [13]. Based on the development needs of new power systems, this review focuses on the material preparation and application of FGM. Meanwhile, we summarize advances in thermal conductivity enhancement and functional innovation of FGM. Finally, prospects for smart early warning and condition monitoring in cable accessories are discussed.

## 2. Materials Research in FGM

FGM exhibit tunable electrical, dielectric, thermal and even mechanical properties under applied electric fields. Semiconductive or conductive particles are normally used as fillers. The mechanism primarily originates from the (dynamic) formation of filler networks, interfacial polarization responses, and carrier transport behavior. In nonlinear conduction paths, the current flow direction does not coincide with the electric field vector, which leads the carrier transport paths to be disordered. The conduction mechanism follows the interfacial principles of the conducting particles [14].

For this chapter, the literature search was guided by the consideration that the matrix and fillers are the core determinants of the nonlinear conductivity of FGM. Accordingly, the matrix types, filler loading, filler species, hybrid formulations, and surface modification strategies were systematically reviewed.

### 2.1. Selection of Polymeric Matrix

The most widely used matrices for FGM include epoxy resin (ER), Ethylene Propylene Diene Monomer (EPDM) and silicone rubber (SR).

**Figure 1:**
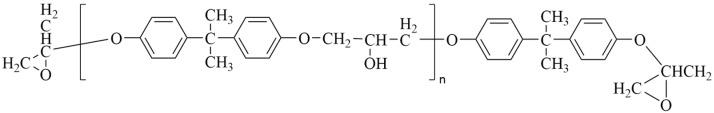
The structural formula of bisphenol A-type epoxy resin (DGEBA).

ER enables the production of products with varying properties through the design of reactive monomers. It exhibits significant development potential due to its excellent corrosion resistance, thermal stability, and mechanical properties, making it suitable for preparing FGM [15]. By modifying ER through the incorporation of functional fillers such as SiC or ZnO, its nonlinear conduction characteristics can be tailored. This allows the threshold electric field and nonlinear coefficient to be adjusted to meet the requirements of different electrical equipment [16,17]. The molecular structure of ER typically contains epoxy groups, as shown in Figure 1, with the bisphenol A-type epoxy resin being the most common, which can serve as the matrix materials for FGM [18]. The ER matrix can meet the demands of medium- and high-voltage cable accessories. For example, G-20 epoxy resin cold-cast compounds are suitable for cable terminations and joints rated for 1~10 kV. According to the IEC 60859 [19] standard, ER bushings are used for the external insulation of gas-insulated switchgear cable terminations, applicable at voltage levels up to 110 kV [20,21].

**Figure 2:**
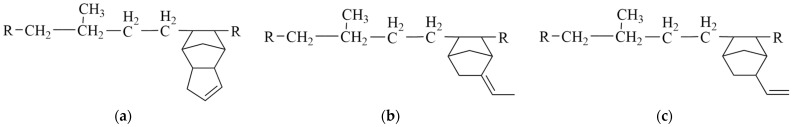
Molecular formulas of EPDM with different tertiary monomers. (**a**) ENB-type EPDM, (**b**) VNB-type EPDM, (**c**) DCPD-type EPDM.

EPDM is a terpolymer formed by copolymerizing ethylene, propylene, and a non-conjugated diene monomer. The content of the third monomer is usually very low, resulting in a highly saturated main carbon-chain structure without polar substituents. Its molecular chain is highly flexible, and it exhibits excellent insulation, corrosion resistance, ozone resistance, aging resistance, and low-temperature performance [22,23]. EPDM matrix is widely used as insulation and sheathing material. Such FGM are commonly applied in medium- and low-voltage cable equipment. IEC 60502-4 [24] requires cable accessories to pass water-immersion tests, heating cycle tests, and AC/DC withstand voltage tests. EPDM cold-shrink products, which require no open flame during installation and provide lasting sealing, are listed as recommended materials. IEC 61442 [25] provides test-method benchmarks for cable accessories rated 6 kV to 30 kV, and EPDM cold-shrink terminals generally comply with these standards [26,27]. The structural formulas of these monomers incorporated into EPDM are shown in Figure 2.

**Figure 3:**
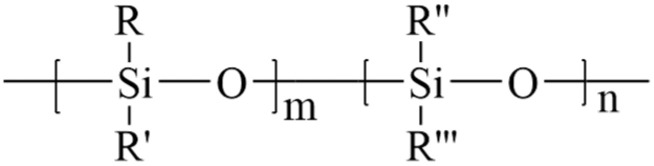
Molecular structural formula of silicone rubber, R, R′, R″ and R‴ represents organic groups such as methyl, phenyl, vinyl, and trifluoropropyl respectively.

Attributed to a low unsaturation molecular structure, as shown in Figure 3, SR outperforms EPDM in terms of high- and low-temperature resistance, electrical insulation, aging resistance, high chemical stability, hydrophobicity, and chemical stability. Testing items cover key electrical and environmental performance indicators, including breakdown voltage, dielectric loss, and aging resistance. Chinese testing standard, GB/T 20779.2-2007 [28], Rubber for electric power safety—Part 2: Rubber for cable accessories, explicitly lists SR as the core material for medium- and high-voltage cable accessories, such as stress cones and cold-shrink tubes. For example, the model FXBW4-220/160 suspension insulator is used in high-voltage transmission lines and is applicable to 110 kV to 500 kV systems, the HY5W8-17/50 outdoor lightning arrester employs overall SR encapsulation [29,30,31].

### 2.2. Aging of FGM

Considering the long-term operational stability of cable accessories, the aging of rubber materials warrants significant attention. Environmental factors such as heat, oxygen, ozone, light exposure, mechanical stress, and chemical media, as well as temperature and humidity, can substantially affect the performance of rubber. For instance, high temperatures accelerate the thermal aging of rubber, leading to a decline in elasticity and strength, while low temperatures can cause rubber to harden and become brittle. Prolonged exposure to ultraviolet radiation can result in the scission of rubber molecular chains, thereby degrading its performance.

Changes in the macroscopic properties of materials often originate from profound alterations in their microstructure. Taking SR as an example, under thermo-oxidative conditions, the side groups of the SR molecular chains break off, while new Si–O–Si crosslinking points continuously form between the main chains. This leads to a progressive increase in the material’s crosslinking density and a gradual loss of its organic characteristics. Research by Su et al. [32] shows that with prolonged thermal aging time, the macroscopic electromechanical properties of silicone rubber undergo regular changes: hardness and elastic modulus gradually increase, while tensile strength and elongation at break decrease significantly, indicating that the material tends to become hard and brittle. Simultaneously, the breakdown strength increases with aging, but the rate of increase gradually slows and the data scatter expands. EDX and ATR-FTIR analyses reveal that during the aging process, the relative carbon content in the silicone rubber system decreases, the relative oxygen content increases, the characteristic absorption peak of Si–O–Si intensifies, while absorption peaks corresponding to organic groups such as Si–CH_3_ weaken. This microstructural evolution dominated by crosslinking is the fundamental reason for the macroscopic hardening, embrittlement, and loss of flexibility of the material. It clearly reveals the chemical and physical mechanisms behind the performance degradation of SR in cable accessories.

Common preventive measures include the use of aging-resistant materials [33], the addition of antioxidants [34,35], and the optimization of formulations [36,37] and processing techniques [38] to mitigate rubber aging. Furthermore, the degradation of molecular chains and the consequent reduction in organic content lead to a decline in the material’s mechanical, physical, and thermal properties. This directly weakens intimate interfacial contact, thereby increasing interfacial thermal contact resistance and potentially inducing new interfacial defects under high electric fields. Yu et al. [39] studied the thermal aging of semiconductive silicone rubber used in 10 kV cold-shrink accessories. Their work showed that as aging temperature and duration increased, the initial shear modulus of the material dropped significantly, surface roughness increased, and organic functional groups such as Si–CH_3_ and Si(CH_3_)_2_ underwent scission and oxidation. These changes manifest as a reduction in macroscopic mechanical performance, i.e., a decrease in interfacial pressure within cold-shrink accessories. Such deterioration in mechanical and thermal properties caused by molecular chain degradation and loss of organic content directly impairs tight interfacial contact, raises interfacial thermal resistance, and may initiate new interfacial flaws under high electric fields. Therefore, before optimizing interfaces through surface modification and structural design, suppressing the intrinsic aging of the material itself is a prerequisite for maintaining low interfacial thermal resistance and long-term operational reliability.

**Figure 4:**
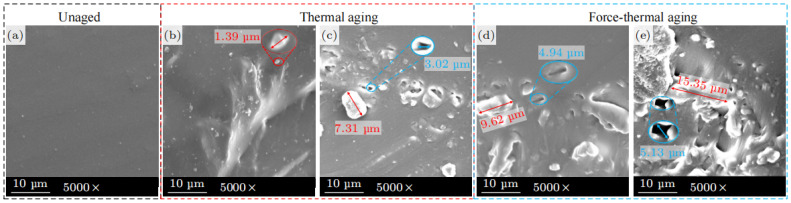
Cross-sectional microstructure images of aged SIR specimens: (**a**) Unaged 0 h; (**b**) thermal aging for 720 h; (**c**) thermal aging for 2160 h; (**d**) force–thermal aging for 720 h; (**e**) force–thermal aging for 2160 h [40].

Represented by SR, electrical insulating materials face aging issues under the coupling of multiple physical fields such as heat and mechanical stress during long-term operation. Force–thermal combined aging causes more severe damage to SR than thermal aging alone, accelerating the deterioration of its electrical and mechanical properties. Li Guochang et al. [40] systematically studied the performance evolution of SR used in cable accessories under thermal aging and force–thermal combined aging, as well as its impact on the internal multi-physical field distribution of accessories, through a combination of experiments and simulations. As shown in the microstructures in Figure 4, the cross-sectional morphology of SIR without aging is smooth, flat, and free of voids. With increasing duration of both thermal aging and force–thermal aging, aggregate precipitation occurs, accompanied by the appearance of voids. However, force–thermal combined aging causes more significant micro-defects than thermal aging alone, including the precipitation of larger aggregate blocks and the formation of numerous voids.

**Figure 5:**
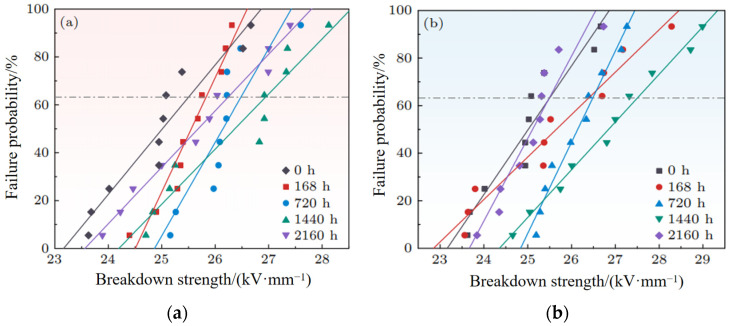
Variation in breakdown field strength of aged SIR specimens: (**a**) thermal aging SIR specimens; (**b**) force–thermal aging SIR specimens [40].

Moreover, the reduction in breakdown field strength caused by force–thermal combined aging is greater than that caused by thermal aging alone, as shown in Figure 5.

### 2.3. Filler Optimization Strategies

When examining the literature of the past five years, it is observed that some fillers can significantly enhance the nonlinear coefficient even at very low contents (<1 wt%), which differs markedly from conventional high-loading strategies. Based on this observation, the concept of “trace co-fillers” is summarized as an emerging design strategy, and a horizontal comparison among single-filler, hybrid-filler, and trace co-filler systems is provided.

#### 2.3.1. Single Fillers

Research on single fillers primarily focuses on aspects such as filler morphology, content, and dispersion state to study key properties of FGM.

Carbon black (CB) is the earliest material used as a nonlinear conductive filler and is still employed today due to its pronounced effect and low cost [41,42]. The widespread use of micron-scale CB is due to its better uniform dispersibility. In terms of morphology, high structure CB (e.g., chain-like) more easily forms a three-dimensional conductive network, enhancing the nonlinear conductivity of FGM. Increasing filler content elevates conductivity and induces a nonlinear abrupt change near the percolation threshold, but excessive filling can impair mechanical performance. Relevant studies are summarized in Table 1.

ZnO and SiC have attracted significant attention due to their pronounced nonlinear conductivity [49,50,51,52]. ZnO is an excellent ceramic material widely used in electrical equipment such as transformers, nonlinear switches, circuit breakers, and cable accessories. Research primarily focuses on utilizing its varistor properties to regulate FGM. Nano-ZnO facilitates achieving strong nonlinearity at low filler loadings but is prone to dispersion challenges, while micron-sized ZnO offers better processability. Special morphologies (e.g., needle-like, tetrapod-shaped) of ZnO readily form conductive pathways, thereby optimizing electrical performance. The filler loading is typically kept below 50 vol% to balance nonlinearity and mechanical properties. Related studies are summarized in Table 2. Common modification strategies mainly include treatments with silane coupling agents to improve interfaces.

The main advantage of ZnO lies in its relatively low cost as a semiconductive filler, while also contributing to the enhancement of thermal conductivity in FGM. Meng et al. [61] prepared ZnO nanosheets via the sol–gel method and doped them into a SiR matrix to investigate the electrical, mechanical, and thermal properties of the ZnO/SiR composite dielectric. The results showed that with increasing ZnO doping content, the nonlinear conduction threshold electric field of the composite gradually decreased, and the breakdown strength reached its optimum value at a doping level of 5 wt%. Meanwhile, the introduction of ZnO also significantly improved the material’s thermal conductivity and mechanical tensile performance, which also matched with their COMSOL multiphysics simulations.

Initially used for stator insulation coatings in electric motors to address corona phenomena, SiC is now employed in both FGM research and production due to its excellent nonlinear conduction characteristics [62,63]. Nanoscale SiC exhibits more pronounced electron confinement and tunneling effects, which helps enhance the nonlinear coefficient, while microscale SiC offers better dispersion and process stability. Beyond conventional granular morphologies, SiC whiskers can form one-dimensional pathways, creating more effective directional conductive or thermal paths within the matrix, thereby improving both electrical and thermal conductivity. Nonlinear behavior typically appears near the percolation threshold at filler contents ranging from 10 to 30 vol%. Related studies are summarized in Table 3.

The advantage of SiC lies in its suitability for high voltage and large-temperature-difference applications, and it is often combined with BN to enhance thermal conductivity. Li Z et al. [69] studied the nonlinear conductive behavior of SiC/epoxy resin materials with different volume fractions (12.25~29.52 vol%) at low temperatures ranging from −196 °C to −46 °C. The results showed that when the filler content exceeded 17.32 vol%, the materials exhibited obvious field-dependent nonlinear conduction characteristics, and the nonlinear coefficient increased with either higher filler content or elevated temperature. Moreover, the freezing effect of carriers at low temperatures significantly influenced the impurity ionization process in SiC, leading to a temperature dependence of conductivity. Gao J et al. [70] investigated epoxy resin materials co-filled with nano-BN and micro-SiC. They found that the addition of BN effectively improved the thermal conductivity of the material and synergistically enhanced the nonlinear conduction characteristics under high electric fields. When the BN content was 2 phr, the highest nonlinear coefficient reached 3.48. Simultaneously, the introduction of BN improved filler dispersion and interfacial compatibility. Its high thermal conductivity helped suppress the negative impact of temperature rise on nonlinear conduction behavior, thereby enhancing the performance stability of the composite over a wide temperature range.

In addition, researchers have also explored the application of other less commonly used semiconductive fillers in the field of FGM to broaden their research and application scope, part of which are summarized in Table 4. Halloysite nanotubes (HNT), as naturally occurring tubular aluminosilicates, had also attracted attention. Hardon et al. [71] incorporated HNT into a two-component cold-curing polyurethane (PU) and prepared composites with 2 wt%, 5 wt%, and 10 wt% HNT. The best performance was achieved at 5 wt% HNT, whose volume resistivity increased nearly 17-fold, permittivity was enhanced by interfacial polarization, and both thermal and mechanical properties improved. Dang et al. [72] studied the microstructure and dielectric properties of BaTiO_3_/epoxy resin materials with different particle sizes. They found that treatment with a silane coupling agent significantly improved the filler–matrix interface, effectively increasing the dielectric constant of the composite while reducing its loss. The study also indicated that filler size and concentration have an important influence on the temperature-dependent dielectric properties. Liu et al. [73] investigated epoxy resin materials with different BaTiO_3_ loadings (0~5 wt%). The results showed that when the BaTiO_3_ loading reached 5 wt%, the electrical conductivity of the composite increased significantly and exhibited obvious nonlinear behavior. Analysis by isothermal relaxation current revealed that as the filler content increased, the number of shallow traps in the material increased and the trap depth decreased, thereby promoting carrier migration and enhancing the nonlinear conduction characteristics of the material. Chi et al. [74] compared the effects of CCTO nanoparticles and nanofibers on the nonlinear conductive behavior of liquid silicone rubber materials. They found that CCTO nanofibers with a high aspect ratio could form more effective conductive pathways even at a low filler content (3 vol%), exhibiting superior nonlinear conduction characteristics and electric field homogenization capability. Han et al. [75], based on the dielectric and micro-morphological characteristics of WS_2_, studied its performance as a nonlinear conductive filler for FGM, providing a new perspective for the selection of nonlinear conductive fillers. The research demonstrated that when the WS_2_ content exceeded 4.31 vol%, the composite displayed clear nonlinear conductive behavior. As the filler content increased, the breakdown field strength decreased and the nonlinear coefficient increased. Furthermore, the introduction of WS_2_ improved the thermal conductivity of the composite by approximately 45% at 25 °C and 40% at 75 °C.

#### 2.3.2. Hybrid Fillers

Building on the study of single-filler systems, the use of two or more fillers in combination can further optimize the nonlinear conductive properties of FGM through synergistic effects. Related studies are summarized in Table 5.

Filler combination, as a common modification method for composite materials, has been studied early on by scholars. For instance, Mårtensson et al. [44], using SiC and CB co-filled EPDM as a model, revealed that combined fillers can synergistically optimize nonlinear conductive properties by regulating the interfacial barriers and percolation networks between different fillers. Their findings on the differences in carrier types (electrons/ions) and activation energy provided a theoretical foundation for subsequent filler selection and interface design. Hu et al. [78] systematically investigated the nonlinear conductive behavior of epoxy resin materials filled separately and in combination with nano-SiC, nano-ZnO, and micro-ZnO. The results showed that when only a single filler was used, the nonlinear coefficient of SiC/EP materials was higher than that of ZnO/EP materials.

However, when micro-ZnO and nano-SiC were combined at a mass ratio of 2:3, the nonlinear coefficient of the composite increased significantly to 3.506, representing improvements of approximately 0.82-fold, 2.48-fold, and 5.01-fold compared to single-filler SiC/EP, micro-ZnO/EP, and nano-ZnO/EP materials, respectively. The study pointed out that micro-ZnO, with its aspect-ratio advantage, readily forms conductive pathways in the matrix, while nano-SiC contributes high conductivity. Their combination creates more effective carrier migration pathways at the interfaces, thereby synergistically enhancing the nonlinear response of the composite. Building on this, Chi et al. [82] further constructed AgNPs/BN composite fillers by growing silver nanoparticles (AgNPs) on the surface of hexagonal boron nitride (BN).

#### 2.3.3. Trace Co-Fillers

Building upon research on single filler and combined filler systems, trace co-filler systems have gradually attracted attention to further reduce filler loading while enhancing nonlinear conductive performance. Their characteristic lies in using other trace amounts of fillers in addition to a primary filler to jointly improve the properties of the composite. The main difference from the combined fillers discussed earlier is that the amount of trace co-fillers is extremely small, typically with a net content not exceeding 1 wt% or differing by more than 10-fold from the loading of the primary filler, yet they can still significantly improve the nonlinear conduction and other characteristics of FGM. Selected typical studies are summarized in Table 6. The research progress on trace co-fillers will be reviewed below in terms of material selection, modification strategies, and performance.

Balancing the filler loading has always been a challenging issue; excessive filler disrupts the overall interface, while insufficient filler fails to significantly enhance the nonlinear electrical conductivity (the enhancement exceeds one order of magnitude). The advantage of trace co-fillers lies in achieving a notable performance leap at low loading levels, while also helping to preserve the original mechanical, physical, and insulating properties of the matrix. Chi et al. [83] prepared Fe^3+^ doped ZnO nanoparticles (Fe_x_Zn_1−x_O NPs) via a sol–gel method and used them as fillers to fabricate EPDM-based materials. The results showed that appropriate Fe^3+^ doping could significantly increase the nonlinear coefficient of the composite at a relatively low filler content while also raising the breakdown field strength. With Fe_0.03_Zn_0.97_O NPs at a loading of 6.32 vol%, the nonlinear coefficient increased by about 10% compared to undoped ZnO/EPDM, and the breakdown field strength improved by more than 120%. Chi et al. [84] investigated the effect of co-doping with trace silver particles and hexagonal boron nitride on the properties of EPDM materials. When 0.5 wt% Ag was added alone, the nonlinear coefficient of the composite increased to 2.45, but the breakdown field strength dropped sharply from 137.1 kV/mm for pure EPDM to 70.5 kV/mm. Therefore, 10 wt% BN was introduced simultaneously to form an Ag/BN/EPDM composite system. While maintaining good nonlinear conduction (nonlinear coefficient of 1.90), the characteristic breakdown field strength was raised to 109.7 kV/mm, representing a 55.6% improvement over the single-Ag-filled system. Moreover, the tensile strength and elongation at break of the material were also significantly enhanced.

**Table 6:** Research and innovation on combined trace co-fillers for nonlinear conduction.

Main Filler			Modification Strategies	Trace Co-Fillers			Matrix	References
Filler	Size & Morphology	Loading		Filler	Size & Morphology	Content		
BN	300~500 nm, flaky	10 wt%	Blending KH550	Ag	100~200 nm, granular	0.2~0.5 wt%	EPDM	[84]
ZnO	200~300 nm, Irregular flaky	5 wt%	Blending	MWCNTs	50~70 nm, Smooth Tubular	0.03~0.1 wt%	SR	[79]
WS_2_	50~100 nm, flaky	23 wt%	Blending Mixed-acid treatment	MWCNTs	10~30 μm, Fibrous	0.19~1.11 wt%	EPDM	[85]
WS_2_	50~100 nm, flaky	23 wt%	Blending H_2_O+IPA	Ionic Liquid	—	0.19~1.11 wt%	EPDM	[75]
ZnO	100~200 nm, flaky	10 wt%	Blending	Fe_0.01_ZnO_0.99_ Fe_0.02_ZnO_0.98_ Fe_0.03_ZnO_0.97_	100~200 nm	1.12~6.32 vol%	EPDM	[83]
BN	5~10 μm, flaky	10 wt%	Melt blending KH550	AgNPs	20~40 nm	0.05~0.35 wt%	EPDM	[82]
SiC	10 μm, particles	34.0 wt%	KH550	BN	100 nm, lamellar	0.97 wt%	EP	[70]
Al_2_O_3_	150 nm, particles	28.2 wt%	Melt blending	Graphene	7~12 μm, 2D Lamellar	0.18 wt%	SR	[86]

Building on this, Han et al. [85] combined multi-walled carbon nanotubes (MWCNTs) with WS_2_. By adding a very small amount of MWCNTs (0.19~1.11 wt%), they significantly enhanced the nonlinear conduction characteristics of the WS_2_/EPDM composite at 25 °C while also improving the material’s thermal conductivity. Nie et al. [87] employed electroless silver plating on tetrapod-shaped ZnO whiskers (T-ZnO@Ag) to fill silicone rubber. At a filler content of only 2.4 vol%, the nonlinear coefficient reached 10.95. The percolation threshold was far lower than that of traditional spherical ZnO fillers (~39 vol%), and the nonlinear conduction characteristics were also markedly strengthened.

The above studies demonstrate that trace co-fillers can significantly enhance the electrical properties of materials with extremely low loadings while simultaneously balancing thermal and mechanical characteristics, such FGM are expected to play an important role in cable accessories. However, existing research has largely been focused on systems with a single trace filler, while exploration of the combined effects of multiple trace fillers remains relatively limited.

#### 2.3.4. Structural Optimization

In addition to regulating the properties of materials by means of filler type, compounding and trace addition, structural design and surface modification of the fillers themselves are also important approaches to improving the comprehensive performance of electric field-regulated materials. Structural optimization mainly includes filler surface modification, morphology control, and oriented arrangement in the matrix, aiming to improve the interfacial compatibility between fillers and the matrix at the microscopic level, construct efficient conductive and thermally conductive networks, and thereby synergistically enhance the electrical, thermal and mechanical properties of the materials. Surface treatment of fillers with silane coupling agents is the most common method, as summarized in Table 7. This section systematically summarizes the current common structural optimization strategies and their effects on the properties of materials.

Silane coupling agents are common chemical additives widely used at inorganic–organic interfaces. By pretreating the filler surface or directly adding them to the matrix material, a bonding layer of organic matrix–silane coupling agent–inorganic filler can be formed at the interface through the reactivity or compatibility of their functional groups. This improves the dispersion of fillers in the resin matrix and further enhances the mechanical, electrical and weather resistance properties of materials. Relevant explorations have also been conducted in the research of FGM. Zhao et al. [60] functionalized the surface of ZnO varistor microspheres using 3-aminopropyltriethoxysilane (KH550), which significantly improved their interfacial compatibility with the silicone rubber matrix. The results showed that, compared with the untreated system, the tensile strength of the surface-modified composite increased by more than 20%, the thermal conductivity was significantly improved, and stable nonlinear conductivity was maintained under 50% tensile deformation. On this basis, Chi et al. [65] modified silicon carbide whiskers (SiCw) with KH550, KH560 and KH570 respectively, which effectively improved their dispersion and compatibility in epoxy resin. The nonlinear coefficient of the 2 vol% SiCw/EP (1% KH550) composite increased from 1.25 for the unmodified filler to 3.03. Among them, KH550 modification yielded the best filler dispersion and matrix compatibility, and the conductivity of the composite dielectric showed the strongest dependence on electric field strength. The breakdown field strength of the 3 vol% SiCw/EP (1% KH550) composite dielectric increased from 16.14 kV/mm for the unmodified sample to 46.37 kV/mm.

On the basis of surface modification with silane coupling agents, Ruan et al. [91] successfully prepared BN/PMIA composite papers via a filler size-matching strategy to simultaneously improve the thermal conductivity and insulating properties of insulating materials. In this study, micron-sized and nano-sized BN were surface-modified with silane coupling agents, which effectively solved the problem of nano-filler loss during preparation. The results showed that when the filler ratio was 12 wt% micron-sized BN and 3 wt% nano-sized BN, the thermal conductivity of the composite was increased by 205% compared with the matrix; when the ratio was 3 wt% micron-sized BN and 12 wt% nano-sized BN, the breakdown field strength was increased by 48%.

Micron-sized and nano-sized fillers are commonly used filler sizes. Generally speaking, the smaller the filler particle size, the lower the current density of the composite under high electric fields and the more significant the improvement in breakdown field strength. However, nano-sized fillers tend to significantly reduce the nonlinear coefficient of the material, whereas micron-sized fillers can maintain or even slightly improve the nonlinear characteristics. The generally accepted reason is that nano-sized fillers introduce deep traps due to their strong interfacial effects, which suppress carrier migration and weaken the nonlinear response, while micron-sized fillers are dominated by shallow traps and have less influence on nonlinear conductivity [92,93].

At the microscopic level, the inherent limitations of materials can be overcome by controlling the oriented arrangement of fillers using external-field-assisted methods, such as electric fields, magnetic fields, stretching, or shear flow fields. Wu et al. Reference [94] successfully realized the oriented arrangement of micron-sized BN and nano-sized Al_2_O_3_ in a polydimethylsiloxane (PDMS) matrix by applying an electric field. The chain-like thermally conductive pathways formed by the two different fillers under electric-field induction increased the thermal conductivity of the ternary composite to 0.228 W/m·K after orientation, which was significantly higher than that of the randomly distributed state (0.215 W/m·K) and single-filler systems. While this ordered microstructure improved thermal performance, it also optimized the dielectric constant of the composite to 6.6 and maintained a low dielectric loss (<0.1).

Thickness design is generally used to optimize the insulation layer and stress cone of cable accessories. In contrast, microstructure regulation and surface activity modification via coupling agents can simultaneously improve the insulating, conductive, and thermal conductive properties of FGM. The synergistic effect of these three structural optimization strategies achieves a balanced performance, which is of great significance for the design of a series of electrical equipment such as cable accessories for new-type power systems.

## 3. Application of FGM

This paper focuses on the engineering applications of FGM. Given that the primary application scenarios of FGM lie in the electrical domain, the literature search was mainly conducted in the two directions of power systems and power electronics. For power systems, the application status of FGM is systematically reviewed along the three links of generation, transmission, and consumption. For power electronics, applications (e.g., IGBT module encapsulation) have been added to better align with the demands of new power systems for advanced electrical materials.

### 3.1. Application of FGM in Power Systems

#### 3.1.1. Transmission Segment, Electric Field Homogenization and Insulation Enhancement in Cable Accessories

With the rapid development of new energy sources such as offshore wind and photovoltaic power generation, the demand in power systems is also increasing. High-voltage direct-current (HVDC) cables have attracted significant attention to meet the requirements of long-distance, large-capacity, and low-loss power transmission, and the stability of cable accessories is even more critical [95,96,97].

In the area of electric field homogenization and insulation enhancement for cable accessories, Li et al. [98] demonstrated that calcium-copper-titanium oxide (CCTO) filled EPDM materials, as nonlinear field grading materials, can effectively optimize the internal electric field distribution of accessories. CCTO micron-sized fillers prepared by the sol–gel method were uniformly dispersed in the EPDM matrix, and their nonlinear conduction characteristics strengthened with increasing filler content while the threshold field strength decreased correspondingly. Finite-element simulation analysis confirmed that applying a 15 wt% CCTO/EPDM composite to the stress-cone region of an HVDC cable termination significantly suppressed electric field concentration, reducing the maximum field strength at the stress-cone root from 45.4 kV/mm for pure EPDM to 2.37 kV/mm. This effectively avoids surface creeping discharge and enhances the insulation reliability and long-term operational stability of cable accessories.

In AC cable terminations, the use of field grading materials for manufacturing stress cones can further optimize the electric field distribution. Zhao et al. [99] prepared ZnO/SR materials with different filler volume fractions and particle sizes, and established a finite-element model of a 500 kV AC prefabricated rubber stress cone. They systematically analyzed the regulating effect of nonlinear materials on the electric field at the stress-cone surface and its junctions. The results showed that when a nonlinear material with a switching electric field Eb of approximately 1000 V/mm was selected as the insulation layer of the stress cone, the maximum electric field on the stress-cone surface decreased from 10.9 kV/mm (for a fixed-conductivity material) to 3.3 kV/mm, a reduction of about 200%. At the same time, electric field concentration at the interface between the insulation layer and silicone oil was significantly suppressed.

#### 3.1.2. Substation Segment, Electric Field Optimization in Gas-Insulated Switchgear

Gas-insulated switchgear (GIS) is widely used in modern substations owing to its high reliability and compact footprint [100,101]. To address the challenges of electric field concentration and surface charge accumulation on GIS insulators, research is shifting from single-material modification toward multidimensional synergistic regulation of dielectric and conductive properties. Ju et al. [102] improved insulation performance by co-optimizing electrode structure and spacer material gradients. Based on the concept of an elliptical gradient dielectric-constant distribution, the FGM spacer already demonstrated a superior electric field distribution compared to a uniform spacer after electrode shape optimization. The fully optimized final model achieved a fundamental improvement in electric field distribution, with quantitative results indicating that the maximum surface electric field strength along the insulator was reduced by approximately 26.5%.

Du et al. [103] proposed a multidimensional functional material (MDFM) scheme that combines a dielectric-constant gradient (ε-FGM) with a surface nonlinear conductive layer (SNCM). This solution achieves adaptive control under all operating conditions. Under DC steady-state conditions, its nonlinear characteristics adapt to different high-temperature environments, significantly homogenizing the electric field. Under harsh transient conditions such as polarity reversal, MDFM avoids the electric-field enhancement caused by residual charges that would occur with a single SNCM, demonstrating critical synergistic advantages. Moreover, under combined stresses such as DC-superimposed with impulse voltage, it maintains excellent homogenization performance.

#### 3.1.3. Power Consumption and Power Conversion Segment, Optimization of Electrical Machine Equipment

In the power consumption and power conversion segment, the insulation reliability of electrical machine equipment, especially high-voltage generators, is crucial. Severe electric field distortion at the end regions of generator stator coils can easily trigger partial discharges and corona aging. Therefore, developing anti-corona materials with self-adaptive field-grading capabilities has become a key research focus. Nanomaterials based on epoxy resin (EP) have attracted attention due to their tunable nonlinear conduction characteristics.

Liu et al. [104] introduced nano-SiC and organic montmorillonite (O-MMT) into a micro SiC/EP system to prepare O-MMT/SiC/EP materials. This material not only increased the glass transition temperature (up to 85.3 °C) but also significantly enhanced thermal stability. The nonlinear coefficient showed only small fluctuations after aging (maximum 1.465, minimum 1.382), demonstrating excellent resistance to thermo-oxidative aging, making it suitable for long-term insulation protection in high-voltage electrical machines.

Yang et al. [105] modified epoxy resin (EP) with polyaniline (PANI) and carboxylated multi-walled carbon nanotubes (MWCNTs) to prepare PANI-MWCNTs/EP materials. The study found that the introduction of PANI significantly regulated the nonlinear conduction behavior of the composite; it not only increased the nonlinear coefficient (up to 5.32) but also synergistically improved the breakdown strength of the material, achieving a 106.16% enhancement compared with pure MWCNTs/EP. This realized dual optimization of electric field self-adaptation and insulation reliability.

### 3.2. Applications of FGM in Power Electronics

In the field of power electronics under the background of new power systems, the blocking voltage of high-power semiconductor devices, such as insulated gate bipolar transistor (IGBT) modules, has been continuously increasing in recent years. Higher performance packaging materials are needed to support the realization of higher voltage power modules. Among them, packaging materials need to have excellent electrical insulation properties to increase the working voltage of power modules and enhance their reliability [106,107,108].

In power electronic module packaging, electric field concentration easily occurs at the triple point (the junction between the ceramic substrate, metallization, and encapsulation material) at the metallization edge of the DBC substrate. This triggers partial discharge and insulation failure. Diaham et al. [109] used an electrophoresis process with diglycidyl ether of bisphenol F (DGEBF) thermosetting epoxy resin as the matrix, crosslinked with an amine hardener, and added high-permittivity nano-scale SrTiO_3_ particles. They prepared an FGM with a permittivity gradient. This material can form a high-permittivity layer in the high-voltage electrode tip region, thereby effectively mitigating electric field distortion. Under a voltage of 6.5 kV, the FGM structure reduces the peak electric field at the triple point by more than 50%. AC breakdown tests further confirm that the breakdown voltage of the DBC substrate encapsulated with FGM increases from 13–20 kVrms for neat epoxy to 25–33 kVrms. This represents an improvement of about 30% compared to the homogeneously dispersed composite.

Power electronic converters (PECs) based on wide-bandgap (WBG) semiconductors require higher voltage levels and switching frequencies. The insulating materials of WBG modules may suffer from extreme electric field stress due to their increasing blocking voltage and compact size, which triggers partial discharge (PD). High dv/dt, high switching frequency, and high-temperature operation inside the module further aggravate this situation [110]. Faruqe et al. [111] prepared a FGM using 10 μm ZnO as filler and liquid silicone rubber as the matrix. They applied it to an insulated metal substrate (IMS) to suppress PD issues in WBG power modules. Finite-element analysis was performed according to the IEC 60664-1 [112] standard by applying a 60 Hz, 7.7 kVrms sinusoidal AC voltage. The results show that, compared with traditional neat silicone rubber encapsulation, covering the triple point of the high-voltage electrode with the FGM layer reduces the maximum electric field from 86.63 kV/mm to 27.82 kV/mm, a reduction of 67.9%. Experimental characterization shows that when the filler concentration exceeds the percolation threshold, the material exhibits obvious nonlinear conductivity characteristics, with a switching electric field of about 3.3 kV/mm.

## 4. Performance Enhancement and Functional Innovation of FGM

Based on the introduction of current major application scenarios of FGM presented above, it can be seen that FGM have important applications and demands in fields such as power systems, aerospace, defense and military industry, rail transit, microelectronics, electrical equipment, and electronic devices. This poses new challenges and requirements for the performance and functionality of FGM.

For thermal conductivity, considering that new power systems require electrical materials to maintain high insulation while possessing excellent thermal conductivity, the literature was re-examined from the perspective of “high-thermal-conductivity insulation”. Classical thermal conduction theories (phonon transport, interfacial thermal resistance, etc.) were reorganized.

### 4.1. Thermal Conductivity Enhancement

Improving the thermal conductivity of insulating materials can significantly enhance equipment efficiency and service life. For example, a 50% increase in the thermal conductivity of the main insulation can raise the efficiency of an air-cooled generator by 20% [113]. Although extensive research has been conducted since the late 1990s, a systematic theoretical consensus has not yet been established, and the performance of industrial products still lags behind the advanced international level [114,115].

#### 4.1.1. Theoretical Basis

As the matrix of FGM, polymer insulating materials are saturated systems with no free electrons, and the primary heat carriers are phonons. The heat transfer rate depends on the vibrations of molecular chain segments, atoms, and functional groups. Reducing crystal defects and enhancing lattice order can lower phonon scattering and improve thermal conductivity. However, polymers are difficult to form sufficiently ordered crystalline structures; the presence of large amorphous regions and defects leads to severe phonon scattering, resulting in low thermal conductivity. Modifying the molecular chain structure or adding high-thermal-conductivity fillers can improve thermal conductivity [116].

The intrinsic thermal conductivity of a polymer is closely related to its crystallinity, orientation, degree of polarization of polar groups, number of atoms per unit volume, chemical bond strength, branch chain length, and crosslinking density [117,118,119,120,121,122,123,124]. The heat transfer efficiency of a composite depends on the intrinsic thermal conductivity of the matrix and filler, the interfacial structure, and the synergistic effects. The thermal resistance between filler particles is usually low, and constructing an interconnected thermal conduction network enables rapid heat transfer. Therefore, synergistic optimization of the matrix and filler has become a mainstream approach. Taking the BN/EP system as an example, Yan Fuduo et al. [125] systematically reviewed high-thermal-conductivity insulating materials and elaborated the thermal conduction pathway theory, the thermal percolation theory, and the thermo-elastic coefficient theory. These theories are generally applicable to various filled polymer composites.

**Figure 6:**
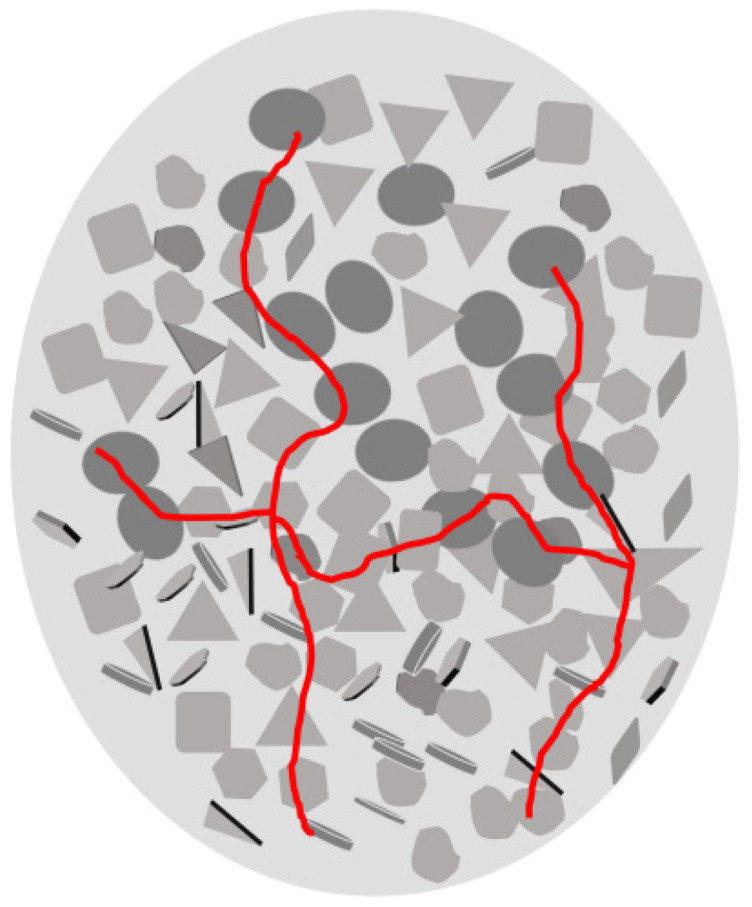
Thermal conduction pathways formed by fillers with different sizes and morphologies.

Taking filled thermally conductive insulating silicone rubber as an example, the thermal conduction pathway theory holds that as the filler content increases, the fillers contact and stack with each other to form a complete thermal conduction network, and heat is transferred along low-thermal-resistance paths, thereby improving thermal conductivity. According to the above theory and research, we can simply draw a schematic diagram according to the heat conduction channels formed by fillers with different sizes and shapes, as shown in Figure 6, the red line can be regarded as the forming of “thermal conduction pathways”. However, the addition of fillers introduces new interfaces, generating interfacial thermal resistance, which in turn hinders the improvement of thermal conductivity [126,127,128].

#### 4.1.2. Improving Thermal Conductivity of Insulating Materials

Generally, there are two main approaches to improving the thermal conductivity of polymers. The first is the preparation of intrinsically thermally conductive polymers through molecular structure design. For example, controlling the liquid crystalline ordered structure of epoxy resin or performing ultra-stretching orientation on polyethylene can form crystal-like arrangements to reduce phonon scattering, thereby increasing thermal conductivity. (For instance, the thermal conductivity of ultra-stretched polyethylene nanofibers can reach ~104 W/(m·K)) [129,130,131]. Huang T et al. [132] adopted a method of modifying the molecular structure to enhance thermal conductivity. They employed a non-solvent-induced phase separation combined with in situ welding strategy to construct a finger-like continuous three-dimensional BN skeleton network in silicone rubber. At a BN content of only about 15 wt%, the in-plane thermal conductivity of the composite reached 15.4 W/(m·K). Theoretical analysis confirmed that this “in situ welding” structure significantly reduced the interfacial thermal resistance between fillers, and the composite exhibited excellent interfacial contact properties, with a contact thermal resistance (<70 K·mm^2^/W) lower than that of most commercial thermal pads. Research indicates that effective general technical routes include the following:, surface functionalization of h-BN (hexagonal boron nitride) through non-covalent and covalent modification methods; preparation of high-performance thermally conductive insulating materials via filler-network design and interface engineering; and oriented alignment and network construction of fillers using external-field alignment, hot-pressing, three-dimensional templating, and other techniques.

Currently, the demand for thermal conductivity in the field of electrical insulation is increasing. Inorganic or organic thermally conductive particles are often used as fillers to prepare high-thermal-conductivity composites. However, the introduction of thermally conductive particles into insulating materials significantly changes their electrical resistance and breakdown strength. The greater the difference in electrical conductivity and dielectric constant between the thermally conductive particles and the polymer matrix, the more severe the internal electric field distortion becomes. High electric field concentration can easily lead to a reduction in the breakdown strength of the matrix. Therefore, when preparing high-thermal-conductivity composite insulating materials, priority should be given to selecting thermally conductive particles that possess excellent electrical insulation, low dielectric constant, low loss, and electrical properties similar to those of the polymer matrix.

On this basis, the second method is to fill the polymer matrix with high-thermal-conductivity fillers (such as BN, Al_2_O_3_, CB, etc.), as shown in Table 8. By controlling the morphology, size, surface treatment, and preparation processes (e.g., electric-field-assisted orientation) of the particles, a thermal conduction network can be constructed and interfacial thermal resistance can be reduced [133,134,135,136].

At the same time, mixing fillers of different sizes or shapes (e.g., whiskers combined with particles) can further optimize thermal conduction pathways [137]. Varlow et al. [138] demonstrated that ZnO/EP materials exhibit significant nonlinear electrical conductivity characteristics when the filler content exceeds the percolation threshold (10~15 vol%). The nonlinearity coefficient increases with higher filler content, and the thermal conductivity of the composite continuously improves as the filler content rises. Meng X et al. [139] reviewed the above methods and pointed out that these approaches not only enhance the thermal conductivity of composites such as epoxy, polyimide, and polyethylene, but also effectively suppress electrical tracking breakdown and heat accumulation, thereby extending insulation life. However, maintaining excellent electrical insulation and mechanical properties while improving thermal conductivity remains a key focus for future research. The approaches to enhance thermal conductivity and the corresponding thermal conductivity enhancement effects of FGM are summarized in Table 9.

**Table 8:** Thermal conductivity and dielectric constant of fillers.

Inorganic Filler	Thermal Conductivity (W/m·K)	Dielectric Constant	Reference
a-BN	0.5~2.0	3.5~5.5	[133]
c-BN	1300	4.2	
h-BN	180	4.2~4.5	
AlN	320	9	[124]
BaTiO_3_	3.5~5.5	—	[132]
Al_2_O_3_	35	9~11	[115]
MgO	40	9.7	
SiC	80~120	9.7	
Si_3_N_4_	180	4.2	
ZnO	30	8.2–11	
MWCNT	3000	—	[140]
Carbon fiber	100	—	[105]
CB	6~174	—	[57]
Graphene	5300	—	[125]

**Table 9:** Thermal conductivity enhancement approaches and effects of FGM.

Matrix	Filler	Approach to Enhance Thermal Conductivity	Enhanced Thermal Conductivity Effect	Reference
CNF	BNNS	Aerogel 3D skeleton template method to construct BNNS/CNF thermal network	50 wt% BNNS, 70 °C, reaches 2.4 W/(m·K), 94.4% increase	[141]
ER	mSiC/BN	Magnetic orientation of SiC+ice-templated orientation of BN to construct double-oriented 3D skeleton	Max 3.35 W/(m·K) at 20 vol%; at 15 vol%, reaches 2.26 W/(m·K), 1312% increase	[142]
ER	APTES modified SiC	Self-assembly of SiC on 3D cellulose aerogel scaffold, followed by vacuum impregnation	At 10.58 vol% SiC, TC = 0.69 W/(m·K), ~300% increase	[143]
EPDM	WS_2_ + trace MWCNTs	Filling with MWCNTs and WS_2_ particles forming bridging thermal pathways	At 1.00% MWCNTs, increased by ~22% compared to 30% WS_2_/EPDM	[83]
ER	SiC ceramic foam	SiC foam forming a 3D co-continuous thermal conduction skeleton	At 15.6 vol% SiC foam, reaches 2.04 W/(m·K), 1000% increase	[144]

#### 4.1.3. Approaches to Enhancing the Thermal Conductivity of FGM

Considering the application scenarios of FGM, there remains a distinction from insulating materials, namely their semiconducting property. Under low electric field strength, FGM must maintain good insulating performance to suppress leakage current. In regions of high electric field, their nonlinear electrical conductivity characteristics primarily serve to homogenize the electric field distribution, thereby achieving adaptive regulation.

**Figure 7:**
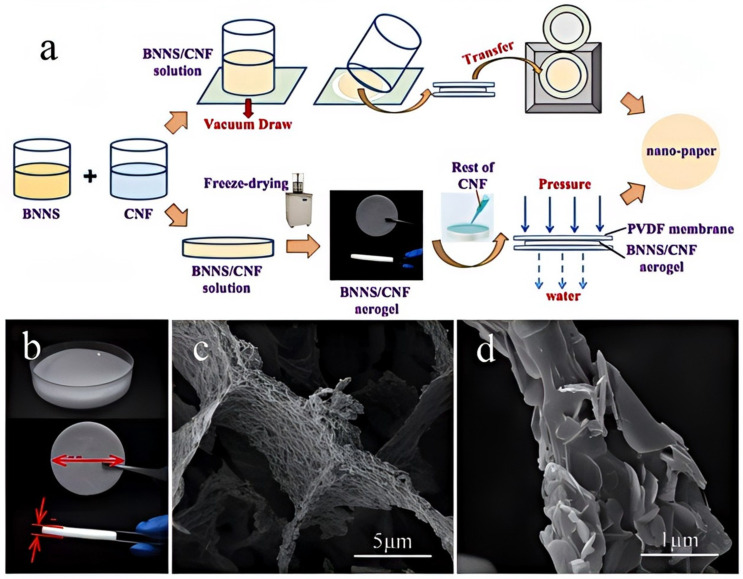
Boron nitride nanosheet (BNNS)/cellulose nanofiber (CNF) composite nanopaper: (**a**) Preparation process of BNNS/CNF, (**b**) images of the BNNS/CNF mixed solution and aerogel, and (**c**,**d**) SEM images of the BNNS/CNF aerogel at different magnifications [141].

The current mainstream approach to enhancing thermal conductivity is to modify the molecular structure at the microscopic level, because this ensures the insulation of polymers while improving thermal conductivity. Wang et al. [141] successfully prepared boron nitride nanosheet (BNNS)/cellulose nanofiber (CNF) composite nanopaper using an innovative aerogel three-dimensional skeleton template method, as illustrated in Figure 7. The core of this approach lies in enabling BNNS to self-assemble and accumulate on the surface of the CNF skeleton, constructing a continuous three-dimensional thermal conduction network. At the same BNNS filler loading, the thermal conductivity of the nanopaper prepared by the aerogel method was significantly higher than that of samples produced by simple blending. When the BNNS content was 50 wt%, the thermal conductivity of the aerogel nanopaper reached 2.4 W/m·K, representing a 94.4% improvement over the 1.2 W/m·K achieved by the blending method. This directly demonstrates that constructing a three-dimensional interconnected thermal conduction pathway is an effective strategy to overcome the performance limitations of traditional blended materials. Furthermore, this BNNS/CNF aerogel nanopaper maintained excellent electrical insulation properties, with a volume resistivity of 4.0 × 10^14^ Ω·cm.

**Figure 8:**
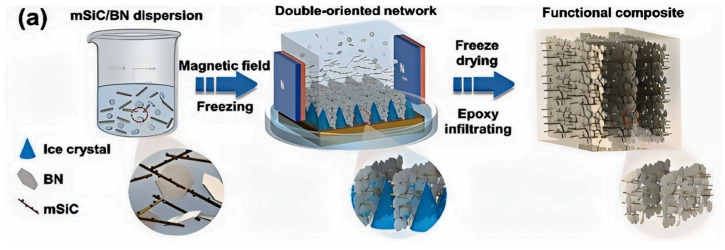

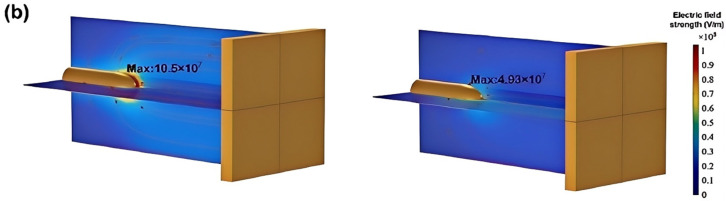
(**a**) Schematic diagram of the preparation and network structure of the cactus-like magnetic SiC/BN composite. (**b**) Distribution of electric field and current density on the needle-type electrode in pure epoxy resin and the dual-aligned composite [142].

Sima et al. [142] designed and prepared an epoxy composite featuring a cactus-inspired dual-aligned SiC/BN network by combining magnetic alignment and ice-templating methods, as shown in Figure 8a. Utilizing the semiconducting properties of SiC and the high thermal conductivity and insulating properties of BN, the magnetically aligned horizontal SiC forms conductive pathways along the electric field direction within the composite. Simulation results indicate this effectively homogenizes the electric field at the electrode tip, reducing the local maximum field strength by approximately 53%, as shown in Figure 8b. Meanwhile, the vertically aligned BN constructs efficient thermal pathways throughout the material. With a filler content of 15 vol%, this composite achieved a 43.7% increase in breakdown strength compared to pure epoxy resin, while its in-plane thermal conductivity reached up to 3.35 W/(m·K), representing an improvement exceeding 1300%.

This work not only demonstrates the improvement in the thermal conductivity of FGM, but also pays special attention to the key indicators of nonlinear electrical conductivity. By combining other relevant studies, we have compared and listed the relationship between the main nonlinear conductivity indicators and the thermal conductivity of FGM in Table 10. It can be seen that these researchers investigated the synergistic improvement of thermal conductivity with key nonlinear conductivity indicators (threshold field strength, nonlinear coefficient) and dielectric breakdown field strength. The improvement in the thermal conductivity of FGM by a single filler is rather limited, even when researchers employ high filler loading or surfactant modification. After the introduction of thermally conductive co-fillers, especially with the construction of three-dimensional thermal conduction paths, the thermal performance of FGM is significantly enhanced. Unfortunately, these studies have so far failed to effectively control the reduction in junction breakdown performance.

**Figure 9:**
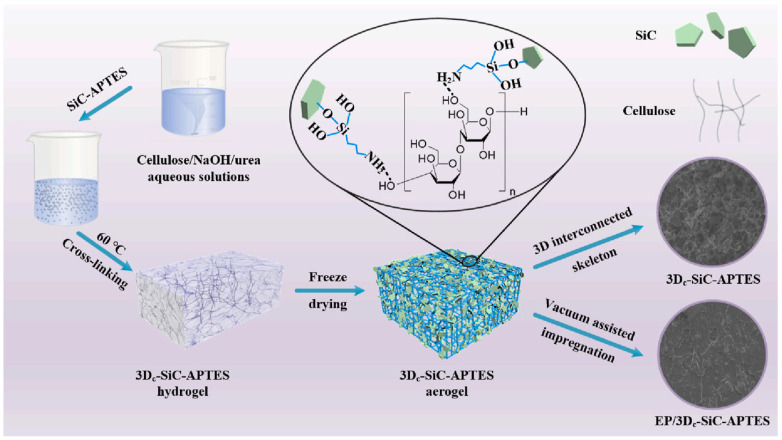
Preparation process of electric field grading materials with self-assembled semiconducting particles on a 3D cellulose aerogel scaffold [143].

Xu et al. [143] constructed a three-dimensional interconnected network by self-assembling surface-modified SiC particles on a 3D cellulose aerogel skeleton, as shown in Figure 9. This structure exhibited nonlinear behavior even at a very low SiC content (4.47 vol%), with a switching electric field of 5.09 kV/mm and a nonlinearity coefficient of 2.17. When the SiC content was 10.58 vol%, the switching electric field decreased to 2.49 kV/mm, the nonlinearity coefficient increased to 4.54, and the thermal conductivity was enhanced by approximately 300%. Simulations further elucidated the mechanism behind the nonlinear field regulation and nonlinearity coefficient for the EP/3Dc graded composite. Additionally, the material’s storage modulus and crosslinking density were significantly enhanced.

### 4.2. Thermochromism

For functional innovation, considering that cable accessories are the primary application scenario of FGM and suffer from high failure rates where traditional monitoring methods have significant latency, thermochromic materials (TCMs) are introduced as a feasible intelligent early-warning solution. When incorporated into the FGM matrix, TCM can achieve reversible color change over different temperature ranges, thereby providing intuitive overheating warning.

#### 4.2.1. Theoretical Basis

Thermochromic materials (TCMs) can reversibly change color with temperature, showing significant potential in areas such as temperature monitoring of electrical equipment and smart energy-efficient buildings. They are mainly classified into four categories: inorganic thermochromic materials (ITCMs), organic thermochromic materials (OTCMs), organic–inorganic hybrid thermochromic materials (OIHTCMs), and polymer thermochromic materials (PTCMs). For ITCMs, the color-change mechanism is due to thermally responsive changes in crystal symmetry upon heating and cooling. The fundamental mechanism involves variations in ligand geometry and/or coordination number induced by temperature changes, which alter the crystal field energy to fall within the photon energy range of visible electromagnetic radiation. For OTCMs and PTCMs, the color-change mechanism is based on changes in molecular structure that lead to variations in the energy levels of the highest occupied molecular orbital (HOMO) and the lowest unoccupied molecular orbital (LUMO) [145,146].

OTCMs and PTCMs are the most sought-after in the field of thermochromic materials due to their excellent color-change performance. Research by Paprota N et al. [147] indicates that introducing different mass ratios of h-BN and c-BN into a thermochromic phase change material based on stearic acid, bromocresol purple, and behenyl alcohol to improve thermal conductivity can enhance its thermal conductivity by nearly 30% while maintaining high phase change enthalpy (approximately 300 J/g) and significant thermochromic performance. This study demonstrates that BN modification can enhance the heat dissipation capability of the composite while also being compatible with other functions such as state visualization. Progress has also been made in integrating intelligent sensing functionality into the material itself, alongside achieving synergistic improvements in thermal conductivity, insulation, and mechanical properties.

In the context of new power systems, thermochromic materials (TCMs) are to be applied in the field of electrical equipment. However, their operating environment is often harsh, including high humidity, high acidity/alkalinity, heavy rain, and heavy snow. Under such conditions, unprotected TCMs are prone to damage and contamination, resulting in diminished or even lost color-change performance. To address this, researchers have improved TCMs through microencapsulation. By forming a stable shell layer on the surface of the active material, the internal substances are isolated from the environment, thereby protecting them and enhancing the stability of the active material [148,149].

**Figure 10:**
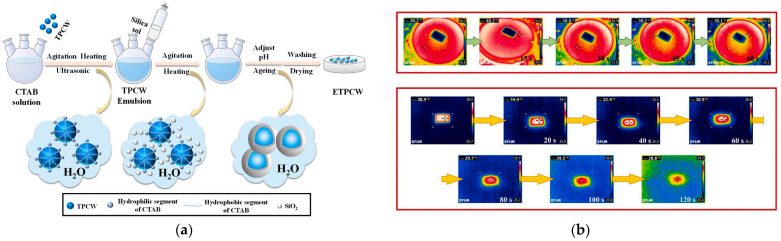
Thermochromic phase change microcapsules materials: (**a**) Preparation process of ETPCW. (**b**) Infrared images of the thermochromic silicone rubber composite (TSRM) at different times during the heating and cooling processes [150].

Zhang et al. [150] prepared thermochromic phase change microcapsules (ETPCW) with paraffin as the phase change core material and silica as the shell layer via an in situ condensation method, as shown in Figure 10a. These were then incorporated into a silicone rubber matrix to construct a composite material possessing dual functionality of temperature self-sensing and thermal regulation, as illustrated in Figure 10b. This material exhibits significant reversible color change (deep blue ↔ light blue/colorless) within its phase change temperature range (approximately 25 °C), possesses a latent heat of phase change of about 130 J/g, and demonstrates good thermal cycling stability. After being applied to a silicone rubber system, the composite maintains excellent hydrophobicity (contact angle >115°) and exhibits mechanical reinforcement.

#### 4.2.2. Approaches for Early Warning in Cable Accessories

The typical failures of cable accessories can be primarily categorized into two types: insulation degradation and overheating failure. Partial discharge is both an early manifestation of insulation faults and a direct cause accelerating the aging of insulating materials. Simultaneously, prolonged overheating at joints leads to irreversible degradation of material properties and is a core factor triggering accessory failures [151,152,153,154].

In the context of new power systems, the data of cable accessories also need to be visualized, requiring more advanced and mature monitoring. Traditional temperature monitoring methods for cable accessories, which rely on manual inspection and fixed-threshold alarms, suffer from problems such as discontinuous monitoring, high false-alarm rates, inability to provide early prediction, and almost no capability for timely intervention. Research on thermochromic intelligent materials within FGM thus holds great application potential in cable accessories. The color-change mechanism based on microcapsule rupture can enable visualization of insulation failures. Thermochromic materials change color with temperature, providing intuitive overheating warnings.

Sima et al. [155] from Chongqing University prepared an insulating material capable of local overheating sensing and early warning for electrical equipment by doping reversible thermochromic microcapsules into an epoxy resin matrix. The microcapsules used fluorane red as the color former, bisphenol A as the developer, hexadecanol as the organic solvent, and melamine-formaldehyde resin as the shell material, with the color-change temperature concentrated between 45 °C and 55 °C. Test results showed that the composite had a mass loss rate below 0.139% after aging at 70 °C for 480 h and could respond to temperature changes within 15–30 s, enabling precise localization of multiple tiny overheating areas.

Building on this, Li et al. [156] prepared reversible thermochromic microcapsules using melamine-formaldehyde resin as the shell to encapsulate a thermochromic core composed of 6′-(diethylamino)-1′,2′-benzofluorane (DCF), bisphenol A, octadecanol, and disperse orange. These microcapsules were then doped into epoxy resin to form an insulating coating with temperature-sensing functionality. The microcapsules had a particle size mainly distributed between 1 μm and 12 μm, with a dense shell. Thermogravimetric analysis indicated extremely low mass loss below 113 °C, demonstrating good thermal stability. The incorporation of microcapsules had a limited effect on electrical insulation performance; the dielectric loss of the composite coating at 50 Hz power frequency was only 0.04–0.05. The material began to change from red to orange when heated to 62 °C and returned to red when cooled to 60 °C. The color difference before and after the change was significant, enabling visual early warning of local overheating in electrical equipment such as cable accessories. Jiang et al. [157] proposed an intelligent sensing material based on reversible thermochromic microcapsules. By doping silicone rubber with these microcapsules at a concentration of 1.5 wt%, the resulting composite material exhibits significant reversible color change at approximately 50°C (with a maximum color difference of 37.24), demonstrating sensitive thermal responsiveness. This material enhances insulation strength by 6.92% while maintaining a hydrophobic contact angle of 115.3° and good thermal aging stability. It offers a new material pathway for realizing visual early warning of overheating states in cable accessories. Notably, silicone rubber is a commonly used insulating material for accessories.

## 5. Summary

This review systematically categorizes the material system, theoretical mechanism, performance regulation, engineering applications, and functional innovation of field grading materials (FGM) for new power systems. The main conclusions are as follows:

In terms of material system, the polymer matrices of FGM are still dominated by epoxy resin, EPDM, and silicone rubber, which are selected according to the temperature resistance, flexibility, and processability requirements of cable accessories and power equipment. The filler strategy has shifted from traditional single fillers (ZnO, SiC, carbon black) to hybrid fillers and trace co-fillers, which can significantly improve nonlinear conductivity at low loading and balance insulation strength and mechanical properties. Surface modification and oriented structure design are effective ways to optimize filler dispersion and interfacial compatibility.

In terms of theoretical mechanism, the nonlinear conductivity of FGM mainly originates from interfacial tunneling, hopping conduction, and Schottky barrier transport under high electric field. The thermal conduction mechanism relies on phonon transport and continuous thermally conductive networks. The coupling mechanism between nonlinear electrical conductivity and thermal conductivity has become the core theoretical basis for performance synergy optimization.

In terms of engineering applications, FGM have been widely used in electric field homogenization of cable accessories, insulation optimization of GIS spacers, and triple-point field strength suppression in IGBT modules, which effectively improve the operational reliability of key equipment in new power systems.

In terms of functional innovation, constructing three-dimensional interconnected thermally conductive networks can greatly enhance the thermal conductivity of FGM and alleviate heat accumulation. The thermochromic microcapsule technology enables visual early warning of local overheating, realizing the intelligentization of FGM.

## 6. Outlook

Although FGM have achieved important progress in laboratory research, there are still prominent contradictions between material performance, long-term stability, and engineering requirements under the complex service conditions of new power systems. Future research should focus on the following directions:

High-performance and stable material systems: Develop low-percolation-threshold, high-nonlinear-coefficient FGM with strong resistance to multi-field coupling aging (electric–thermal–mechanical–chemical), and clarify the long-term performance evolution mechanism.

Multifunctional synergistic design: Break through the synergistic optimization technology of nonlinear conductivity, high thermal conductivity, high breakdown strength, and self-healing/thermochromic intelligence, to achieve integrated functions of electric field regulation, heat dissipation, and status early warning.

Adaptation to new power system scenarios: Develop FGM suitable for wide temperature range, high dv/dt, high-frequency pulsating electric fields, and extreme low-temperature environments, to meet the demands of renewable energy grid connection and offshore/ultra-high-voltage power transmission.

Industrialization and engineering technology: Optimize low-cost preparation processes, solve the problems of filler dispersion and batch stability, carry out long-term operation tests and standard formulation, and promote the large-scale application of FGM in cable accessories, GIS, and power electronic modules.

Intelligent and digital development: Combine with functional materials, artificial intelligence and sensing technology to realize intelligent perception, fault early warning, and life prediction of FGM, supporting the intelligent construction of new power systems.

## Figures and Tables

**Table 1:** Applications and research of CB as nonlinear fillers in FGM.

Size	Morphology	Loading	Matrix Material	References
50 nm	granular	5 wt% 10 wt% 15 wt% 20 wt%	HDPE	[43]
~1 μm	coarse fine particle type	13.1 vol% 11.7 vol%	EPDM	[44]
18~33 nm	granular	10~20 wt%	HDPE	[45]
~20 μm	granular	4.6~7.3 wt%	EP	[46]
—	fine particle	5 vol%	EP	[47]
—	colloidal particles, uniform, crack free	0.4 wt% 0.5 wt% 0.6 wt%	EP	[48]

**Table 2:** Applications and research of ZnO as nonlinear fillers in FGM.

Size	Morphology	Loading	Matrix Material	References
50~300 μm	particles	20 vol%	ER	[53]
20~125 μm	irregular	46.5 vol%	SR	[54]
50~150 μm	microsheres	46.5 vol%	SR	[55]
75~125 μm	grain size	39 vol%	SR	[56]
1 μm	—	15~30 wt%	low-density polyethylene	[57]
120 μm	—	39 vol%	SR	[58]
63 nm	core–shell structure	20 vol%	EPDM	[59]
10~30 μm	microspheres	35~50 wt%	SR	[60]

**Table 3:** Applications and research of SiC as nonlinear fillers in FGM.

Size	Morphology	Loading	Matrix Material	References
0.7~22.8 μm	particles	40 vol%	EPDM	[64]
100~500 nm	whiskers	1~4 wt%	ER	[65]
0.45 μm	uniform particles	30/50/100 wt%	SR	[66]
0.5 μm	particles	10/30/50 wt%	EPDM	[67]
50 nm	uniform particles	25 vol%	SR	[68]

**Table 4:** Innovative applications and research of nonlinear conductive fillers.

Filler	Matrix Material	Filler Size	Morphology	Loading	References
TiO_2_	SR	300 nm	irregular particles	2~8 wt%	[76]
BaTiO_3_	ER	50 nm	particles	5 wt%	[73]
CCTO	LSR	Diameter ≈ 400 nm, Length ≈ 7 μm	granular/fibrous	3~10 vol%/1~3 vol%	[74]
WS_2_	EPDM	200 nm	Multi-layer cluster and thin-layer uniform structure	16.23~36.76 wt%	[75]

**Table 5:** Research and innovation on combined fillers for nonlinear conduction.

Main Filler			Modification Strategies	Combined Fillers			Matrix	References
Filler	Size & Morphology	Loading		Filler	Size & Morphology	Content		
SiC	30~50 nm granular	5 wt%	surface coating with SiO_2_	SiC/SiO_2_	core–shell structure	—	EP	[77]
SiC	30~40 nm granular	3 wt%	melt blending	ZnO	diameter 1.5–2 μm, length 20–27 μm	2 wt%	EP	[78]
PPy	~40 nm spherical	5 wt%	blending, DCP curing	BN	hexagonal, fla-ky	10 wt%	EPDM	[79]
ZnO	~60 nm granular	20 vol%	treatment with SnF_2_/SnCl_2_	SnO	core–shell structure	—	EPDM	[59]
CuNPs	10~30 nm granular	3 wt%	liquid-phase chemical reduction	BN	100~300 nm flaky	10 wt%	EPDM	[80]
BST	~200 nm granular	10 wt%	mix	ZnO	~40 nm granular	10 wt%	SR	[81]

**Table 7:** Research and innovation on structural optimization of nonlinear conductive fillers.

Filler	Morphology & Size	Loading	Matrix Material	Optimization Strategy	Optimization Purpose	References
ZnO	Particles, 2 μm, 10 μm	5 wt%	EP	surface modification with KH560	Improve nonlinear conductivity	[88]
SiCw	Rod-shaped, rough surface, 100~500 nm	1 vol% 2 vol% 3 vol% 4 vol%	EP	surface modification with KH550/KH560/KH570	Improve nonlinear conductivity, suppress breakdown strength drop	[89]
ZnO	Microspheres, 10~30 μm	35 vol% 40 vol% 45 vol% 50 vol%	SR	surface modification with KH550	Improve nonlinear conductivity, thermal conductivity, and interfacial compatibility	[65]
MMT	Lamellar, 161~278 nm	1 wt% 2 wt% 3 wt% 4 wt%	EP	surface modification with KH550 and KH560	Improve nonlinear conductivity, thermal conductivity, and reduce dielectric loss	[60]
ZnO	Rough particles, 2 μm	5 wt%	EP	surface modification with silane coupling agent	Improve nonlinear conductivity, suppress space charge accumulation	[90]
SiC	Granular, ~200 nm	5 wt%				

**Table 10:** Research about synergistic improvement of thermal conductivity, nonlinear conductivity and breakdown strength.

Matrix	Filler	Inflection Point (kV/mm)	Nonlinear Coefficient	Breakdown Strength (kV/mm)	Thermal Conductivity (W/(m·K)	References
SiR	0 wt%ZnO	-	-	158.6	0.140	[61]
	5 wt%ZnO	14.51	2.71	166.3	0.190	
	10 wt%ZnO	10.56	2.81	136.9	0.217	
	15 wt%ZnO	5.96	2.89	118.6	0.219	
EPDM	0~30 phr WS_2_	-	-	148.7~84.1	0.23	[75]
	40 phr WS_2_	18	4.27	86.1	0.33	
	50 phr WS2	13	4.42	66.2	0.31	
	60 phr WS2	7	5.44	65.0	0.28	
SR	35~50 vol%ZnO (untreated)	2.02~1.31	13.0~9.4	-	0.24~0.36	[60]
	35~50 vol%ZnO (KH550 treated)	2.14~1.37	13.9~9.2		0.24~0.38	
EPDM	30%WS_2_/0 CNT	-	-	84.1	0.65	[85]
	30%WS_2_/0.25%CNT	-	-	74.7	0.69	
	30%WS_2_/0.50%CNT	-	-	73.6	0.76	
	30%WS_2_/0.75%CNT	17	-	68.0	0.79	
	30%WS_2_/1.05%CNT	8	-	67.0	0.8	
	30%WS_2_/1.25%CNT	5	-	53.5	0.78	
	30%WS_2_/1.50%CNT	-	-	51.3	0.82	
EP	5 vol%mSiC/BN	-	1.1	14.7	-	[142]
	10 vol%mSiC/BN	10	1.84	16.0	0.69~1.36	
	15 vol%mSiC/BN	8	2.63	20.6	1.17~2.26	
	20 vol%mSiC/BN	4.8	3.27	17.3	3.35	

## Data Availability

No new data were created or analyzed in this study. Data sharing is not applicable to this article.
